# Living with Atrial Fibrillation: A Family Perspective

**DOI:** 10.1155/2022/7394445

**Published:** 2022-03-04

**Authors:** Stine Rosenstrøm, Signe Stelling Risom, Jens Dahlgaard Hove, Anne Brødsgaard

**Affiliations:** ^1^Nursing and Healthcare, Department of Public Health, Aarhus University, Aarhus, Denmark; ^2^Department of Cardiology, Copenhagen University Hospital, Amager Hvidovre, Copenhagen, Denmark; ^3^Department of Cardiology, Copenhagen University Hospital, Herlev Gentofte, Copenhagen, Denmark; ^4^Department of Clinical Medicine, Faculty of Health and medical Sciences, University of Copenhagen, Copenhagen, Denmark; ^5^University College Copenhagen, Institute for Nursing and Nutrition, Copenhagen, Denmark; ^6^Copenhagen University, Faculty of Health and Medical Sciences, Copenhagen, Denmark; ^7^Department of Paediatrics and Adolecent Medicine, Copenhagen University Hospital, Amager Hvidovre, Copenhagen, Denmark; ^8^Department of Obstetrics and Gynaecology, Copenhagen University Hospital, Amager Hvidovre, Copenhagen, Denmark

## Abstract

**Aim:**

The aim of this study was to obtain insights from patients and their family members on how families are living with atrial fibrillation.

**Background:**

Atrial fibrillation is the most common cardiac arrhythmia and is often described as an emerging global epidemic affecting an estimated 33.5 million people worldwide. Living with atrial fibrillation not only affects the patient but also may negatively influence family members' perceived health. The perspective of the family has previously been understudied, and more knowledge on how patients and their family members cope and adjust to life with atrial fibrillation may be helpful when developing future support for patients and their family members when coping with atrial fibrillation.

**Methods:**

A qualitative phenomenological study with an inductive, descriptive research approach based on Giorgi's descriptive method was used. Data were gathered through 12 dyadic family interviews. The COnsolidated criteria for REporting Qualitative research checklist was followed while conducting the study.

**Results:**

Three major themes emerged: *emotional differences*, *changes in family life,* and *uncertainty about the future*. Atrial fibrillation had multiple effects on the family. Frequently, several adjustments and adaptations had to be made to accommodate life with atrial fibrillation.

**Conclusion:**

Patients with atrial fibrillation and their family members feel a need to talk about their emotions and worries. They required support and guidance to manage the challenges of living with atrial fibrillation. These results will be used in a family-focused intervention designed to support families in adjusting and managing their everyday lives with atrial fibrillation.

## 1. Background

Atrial fibrillation (AF) is the most common cardiac arrhythmia and is often described as the new epidemic among heart diseases [1]. It is estimated that, in 2010, 33.5 million people had AF worldwide, with higher incidence and prevalence rates in the developed countries. This number is expected to increase [2]. AF is a complex disease, and its treatment requires a high degree of adherence to prevent complications [3, 4]. Patients may experience palpitations, dyspnoea, and chest pain, and the burden of symptoms often entails impairment of physical and mental performance and poor health-related quality of life [5]. Patients with AF may experience anxiety, feel that they receive too little support and self-management counselling, and often suffer from distress associated with unpredictable symptoms [6, 7]. Managing life with a chronic illness, such as AF, can be distressing not only for the patients but also for their family members [8–10]. In couples where AF is present, a common concern for the spouse is uncertainty related to limited knowledge about AF [11–13]. Patients with AF have also described how they struggle to regain balance, learning to live with a wide range of symptoms after having been diagnosed with AF [14]. Thus, most families need support and guidance in facilitating successful adjustments to cope with this illness [15, 16]. In addition, knowledge about how the family as a system and unit copes with AF in everyday life is lacking. Furthermore, patients and family members facing a chronic illness may have a need and a desire to talk about their beliefs. Typically, family members wish to be involved in the treatment and want information provided by health professionals [17]. Whereas earlier studies have focused mainly on treatment of patients with AF, the last decade has seen a growing interest in such patients' life trajectory after treatment for AF in general and how they experience living with AF in particular [18, 19]. The changes over time might have influenced the experiences of living with AF. Furthermore, studies focusing on the narrative of patients and their family members living with AF are sparse [20]. The present study will further our knowledge about how patients with AF and close family members view life with AF [21–24].

## 2. Aim

The aim of this study was to obtain insights from patients and family members about how families are living with AF.

## 3. Methods

### 3.1. Design

To explore the experiences of patients and their family members, we chose a qualitative study design using a phenomenological approach and an inductive, descriptive research method [25, 26]. Giorgi's descriptive qualitative approach is a way to approach phenomenology, and it is relevant for obtaining in-depth descriptions of lived experiences [27]. The task was to explore and describe objects of experience precisely as experienced by patients with AF and their family members [25]. Descriptive research is, following Giorgi, basic research that describes the phenomenon being focused on [27]. The family interviews were dyadic (two individuals or more), also known as joint interviews [22, 28]. This approach is used in healthcare research to produce data at a family level [22, 29]. Furthermore, dyadic interviews may be used to produce data on the effects of major disruptions caused by chronic illness, not only for the sick person but also for their family members and interpersonal relationships [26]. Interviewing a patient and a family member may produce a beneficial synergistic effect, thereby revealing essential phenomena of the lived experiences of the family unit, which would not be uncovered through individual interviews [29]. Furthermore, we were not interested in identifying the variety of experiences but wished to produce family unit narratives according to the theory of family-focused nursing [30]. The COnsolidated criteria for REporting Qualitative research (COREQ) checklist was followed through developing, performing, and reporting the study [31].

### 3.2. Data Collection and Setting

Data were collected from February to June 2019 through dyadic family interviews with patients with AF. Patients were asked to bring somebody whom they considered to be a close family member [16]. The concept of family was defined as “*family is who they say they are*” [16]. This definition is rooted in the framework of family nursing. Therefore, it was the patient who defined which family member they would bring to the interview, and we did not distinguish between partners and other family members. We informed the patients that it was entirely up to them whom they chose to bring to the interview; it did not necessarily have to be their partner but could be a close friend or neighbour. Furthermore, we chose to limit the interviews to one patient and one family member who had a major role in the patient's everyday life and with whom the patient felt comfortable sharing feelings and thoughts about AF [16]. Patients and families were recruited from an outpatient clinic in a university hospital department of cardiology in the Capital Region of Denmark. The estimated size of the AF population in the outpatient clinic is 400 annual patients with AF. In recruiting families, we used a purposeful sampling strategy to provide rich and varied data material, for example, differences in gender, age, and time since being diagnosed with AF in a family member with AF and the relationship between patient and family member. The inclusion criteria were Danish-speaking patients ≥18 years of age diagnosed with AF regardless of type and treatment. The exclusion criteria were chronic heart failure patients and patients with severe comorbidities. Patients were approached when they met for appointments where nurses from the clinic identified eligible patients and family members accordingly to the inclusion and exclusion criteria. During the recruitment period, 32 patients were asked to participate in the study. Twenty patients declined due to working, not having a family member, or not being interested. However, the recruited patients and the family members represented the variety of the population of the families in the outpatient clinic.

The dyadic family interviews were planned to continue until it was possible to uncover a deeper understanding of the phenomenon of interest, which often occurs with a sample of five to 20 informants [32]. A semistructured interview guide based on a literature search focused on a few broad questions guiding the family interview. The interview guide was only used as inspiration, and the interviewer was always open to any description of the phenomena that could emerge during the interview. The role of the interviewer was to facilitate a situation that would make the patients and their family members reflect upon living with AF and share their experiences through a mutual narrative in order for the researcher to obtain an in-depth understanding of the phenomenon. The family interviews were approached with open questions to stimulate descriptions: “*Can you describe how it is for you to live with one in the family having AF?*” *“How is your everyday life with AF?”* “*How are feelings about AF expressed in your family?*” “*What is important for you to manage everyday life with AF in the family?*” During the interview, the interviewer was aware of both the patient and the family member, and the interview questions were directed towards both by phrasing the questions in a way that allowed both the patient and the family member to describe how they experienced living with AF. This strategy was adopted to obtain insights from the family as a unit by having both parties describe what living with AF was like.

As we had no prior history with the families, the interviewer made a genogram at the beginning of each interview, as recommended by Wright and Leahey [16], to obtain a sense of the family structure. No additional exploration was made as to how the genogram could have added further insights into the family since only one interview was conducted with each family.

The interviews took place according to patients and families' preferences, either in their own homes or in the hospital in a private room a few days after they consented to participate. The interviews were audiotaped and transcribed verbatim by the first author and three nursing students. All transcripts were stored and analysed in Nvivo12 PRO.

Before interviewing the families, the first author, who conducted the interviews, underwent a preunderstanding exam in a peer interview, which revealed experiences of family members with worries that were rarely in focus in the consultations with healthcare professionals, the latter being mainly focused on the medical aspects of AF. The preunderstanding interview was conducted to ensure a nonjudgemental format for conducting the interviews and to ensure trustworthiness by allowing the researcher to use what phenomenology calls “bracketing,” where one's own beliefs about a phenomenon are temporarily suspended in an attempt to avoid influencing both the collection and the interpretation of the data [25]. Total bracketing is impossible in practice, but by being conscious of one's preconceptions, it is possible to keep experiences and prejudices at a distance [32].

### 3.3. Data Analysis

A qualitative methodological approach employing Giorgi's phenomenological method was used to analyse data following four steps [25, 26]. The first step focused on gaining an overall sense of the family unit, using the genograms and notes taken with the families during the interviews, listening to the audiotaped recordings, and reading the entire material several times. The researcher used his/her intuition about and reflection on each transcript. In the second step, “*meaning units*” were identified from the text. Meaning units are small parts of the text that are explicated where a phenomenon emerges. The third step involved regrouping and redescribing statements that were relevant for the meaning units, also focusing on the dyadic narrative of awareness, unawareness, togetherness, and separateness in living as a family with AF [22]. In the fourth and last step, an exemplary narrative was made into a single general analysis, which integrated and synthesised the common and essential aspects of the phenomenon, named themes [26, 27]. The process of analysing data was made in steps 1–3 by the first and fourth authors, who created the preliminary analysis, and disagreements were discussed until an agreement was reached. Step 4 involved the second and fourth authors, who are a senior researcher and a professor, respectively, with expertise in qualitative research. The whole team discussed the essence of the phenomenon until all agreed, thereby enhancing the credibility of the analytical process. The research team agreed to stop including participants because the interviews did not yield new data, no new themes occurred, and the phenomenon was deemed to have been captured sufficiently [33].

### 3.4. Ethical Considerations

All participants received written and oral information about the study, and written informed consent was obtained according to the Helsinki Declaration [34]. Participation was based on willingness, and the participants were informed that data would be safely stored on an encrypted drive and that their identities would be protected when reporting the research findings. Fictitious names were used in the transcribed data. The study was approved by the Danish Data Protection Agency (VD-2019-42) and the local ethical committee (ID: 19007769).

### 3.5. Findings

Four interviews were held in the patients' homes, and eight interviews were conducted in a private room at the hospital. The interviews lasted between 35 and 85 minutes (mean 55 min). Seven women and five men had AF with a mean age of 66 years (range 49–86 years). The family members included seven women and four men, with a mean age of 64 years (range 49–79 years). The family members were primarily spouses (50%). However, one was a sister, one was a daughter, two were male friends, and one was a female friend who accompanied the patient instead of the husband (Table 1). All patients were interviewed with a family member, except for one, who had to cancel the family interview the same day due to commuting issues. Data from the patient interviewed alone were included in the analysis. The point of data saturation was reached after twelve family interviews had been conducted.

The phenomena that emerged from the analysis were captured in the essence of three major themes that described how family life was affected by AF: *emotional differences*, *changes in family life*, and *uncertainty about the future*. The three themes were covered by seven subthemes that emerged from the analysis process (Table 2). The findings show that the essence of living with AF in a family had multiple facets. Still, the families' overall experiences were that despite not being an acute life-threatening disease, AF had multiple effects on emotions in the family, which often led to several adjustments and adaptations of their daily lives.

### 3.6. Emotional Differences

The emotional reactions that patients experienced living with AF often differed from those of their family members. Partners, in particular, appeared to be very concerned, especially in the beginning when their partner was diagnosed with AF. As time went by, the family members became more familiar with AF, the symptoms, and its treatment. There were variations between the families regarding how they talked about AF and the family members' involvement in the challenges that arose in their daily lives. Some patients and their close family members shared very little about their feelings or worries about AF, whereas others shared every single thought about AF in their everyday lives. Some patients explained that they did not share their worries about AF because they did not want to be a burden to their family members or the hospital. Instead, some patients and close family members used their social networks rather than each other to talk about AF.

Female patient: “*I avoided sharing my feelings with him [spouse]. It has to do with the fact that if you know each other very well, you also know how the other person will respond.*” (Family 3)

There was also a desire to protect family members, which occasionally meant that patients did not share all their worries about living with AF. In particular, the female patients explained how they kept their feelings to themselves, and some even preferred to go to the hospital on their own. Several women of all ages felt that talking to the doctor offered a private space for addressing their problems in living with AF. Some patients also felt that their spouses should not take time off from work to go with them to the hospital in order not to let their lives with AF interfere with their spouse's life. However, this was often despite the spouse feeling a need for more information about AF to understand the aetiology behind the arrhythmia.

Male family member: *“Sometimes I worry a lot, and then I try to find some information on the Internet, but it would be better if we had gotten the same information about AF and not… “I have heard something, and you have heard something different.” I believe the personal and individual experience will always be different, whether you are a patient or a family member. Therefore, we need to be able to talk about different questions.”* (Family 6)

The patients and their family members saw their lives after being confronted with AF as being offered a chance to find new meaning in life, for example, taking better care of themselves, minimizing stress, and changing negative lifestyles. Both the patients and their family members expressed that trying to find a new meaning in living with AF often challenged them because of insecurity and a lack of knowledge about symptoms and medication. The patients and their family members described how they were overcoming the challenges in their daily lives and that they felt a need for information about how to live with AF.

Male patient: “*I have had a lot of stress over the years and I have become more and more convinced that my AF is stress-related—and I can still be very busy…. But, especially after the third hospitalization, I needed to get away from it all and understand why.*”

Female partner: “*Yes, it was very hard on the family because I did not understand it all and how I could support him.*” (Family 4)

It could often be challenging for the family to adjust to the new life situation caused by AF. Confusion and lack of knowledge about AF occasionally burdened the patients and their close family members. Most of the family members expressed that they felt that they were more concerned about AF than the patient with AF. Family members experienced how this often led to anxiety and worries in everyday life situations, for example, when going to a party or on holiday. Several close family members were missing information about the causes of AF, and they were often afraid about what might ultimately happen to the patient's heart in everyday situations.

Male partner: *“I am confused about the heart… what about age and physical function… and being overweight? All these things can be a sign that it's dangerous. I'm worried that her heart can do all sort[s] of things or even stop.”* (Family 6)

Patients and family members often had individual perspectives on AF, which could affect their emotions and social interaction with each other living with AF.

### 3.7. Changes in Family Life

Patients and family members across the represented family relationships described how AF brought several changes to their lives. The families used words like guilt if social activities, work, and the household had been downsized due to lacking energy. Some were concerned that their children might get AF, and they experienced changes in their mood if they felt that they were using much energy to find trustworthy information by themselves to understand the nature of AF. The fear of AF symptoms led to insecurity, which influenced both the patient and the family members. The family members experienced that they were dependent on their close ones to obtain information about symptoms on which they could rely to plan daily activities. This insecurity often affected family activities. For example, family members talked about how they could no longer have a meal at a restaurant if the patient with AF had little energy caused by severe palpitations and shortness of breath, forcing them to stay home. Some patients and close family members refrained from social activities and avoided travelling far from home due to fear of sudden rapid AF, which could lead to isolation, and feelings of irritation and guilt would sometimes arise. A few patients claimed to have learned to ignore their AF symptoms in some situations because they did not want the arrhythmia to change their everyday life with friends and family.

A female patient in her sixties described her relationship with her husband whom she chose not to bring to the interview*: “There are some emotions and maybe also irritation – ‘why can´t he [husband] understand that I do not have the energy right now because I feel very low on energy.'”*

Her sister who took part in the interview responded: “*He is very overprotective [the husband], once he stabbed himself with a needle he passed out… he is not good at these things.*”

Female patient*:* “*Maybe I am not good at talking with him [husband] about it. I just go on, even if I am exhausted.*” (All quotes are from Family 8)

None of the family members had prior knowledge of the nature and symptoms of living with AF. They said that they needed knowledge to understand the many aspects of living with AF to support the patient and try to reestablish a normal everyday life without too much interruption or worry.

Daughter: “*I am thinking like this. ‘Could it develop into complications for the heart? Could it suddenly trigger an infarction, or that the heart stops, or could it start an attack?'*” (Family 10)

Some families experienced that their everyday life had become more isolated because they had to limit their social relationships due to reduced energy levels. Furthermore, families described how AF forced them to choose between social activities, either because of a lack of physical and mental energy or to reduce stress to prevent symptoms. Some families felt that the need to prioritise their time and energy for social activities meant that it was very important to be understood by their network of friends.

Male patient: “*I try to be open about what I can and what I can´t do. I once had to cancel a skiing trip. I explained why, and of course my friends understood why, but it was super annoying to cancel.*” (Family 2)

Living with AF had multiple consequences for patients and family members, and patients and their close ones were confronted with the need to implement various changes in their daily lives.

### 3.8. Uncertainty about the Future

The patients and their family members described how they experienced many notions and beliefs about the future living with AF, especially in the first months after being diagnosed with AF. The future that patients and family members imagined included fear of aggravation of AF with complications, such as stroke, and fear of death. The deep inner thoughts and reflections about the consequences of AF were not always discussed between the patients and their family members, and family members talked about feeling hopeless and not knowing how to support their patient.

Male partner: “*In my mind, I have all sorts of beliefs and solutions, but at the same time, it feels like being captured and sometimes you give up.*”

Interviewer*:* “*Do you talk with him about your AF or do you keep your feelings to yourself?*”

Female patient*:* “*No, I do not always talk about it [AF] because it comes and goes.*” (Family 7)

Many family members expressed that they were in a permanent state of alert due to lack of control over when and why AF starts. They were aware that this could be a potential stressor for the relationship in their everyday lives.

The female partner of a middle-aged male patient who had been living with AF for 1.5 years described a situation with her partner: “*So, I do not relax when we are together at a party, and of course I say to myself that now I do not have to monitor the other [husband] … But you can just get a little obsessed and wake up with a feeling of being in a state of alert. I think now, after the third time, it has affected me a lot.*” (Family 5)

Male and close one with AF responds: *“yes it has really affected our family and my wife could really need some support and some information about AF because when I come home from the meeting at the hospital, I don´t remember to give her all that information.”*

Several spouses felt that thoughts of death occasionally arose at night. It was not uncommon for some to watch their partner sleep several times a week. Even though most of the patients and family members knew that AF did not cause cardiac arrest, many felt that they should be prepared for an acute situation that could suddenly occur.

Interviewer: “*Do you observe her at night [female friend]?*”

Male friend to a female patient who had been living with AF for at least two years and with daily symptoms from AF: “*Yes, I actually do… I cannot hear her breathing at all… and when she moves, I notice her pulse… all the time thinking when I*'*m going to do cardiopulmonary resuscitation. I actually think that.*” (Family 8)

Furthermore, most families expressed a need for support from health professionals that could ease their fear and prevent myths about AF. Patients and family members explained how they occasionally had read something about AF on the Internet that made them scared and nervous about what might happen. Family members also described how misinformation would sometimes cause them to be overprotective of their patient with AF in everyday situations. Both the family member and the close one wished to be informed and involved by the healthcare professionals to allow them to provide mutual support for one another in everyday life with AF.

Female friend: “*I think about how my thoughts go spiralling … and all of a sudden, I'm thinking… what can happen? And can this happen, and what about this? Then I need someone to say that I should calm down and tell me that this will not happen, and it´s not like that.*” (family 11)

Male patient and close one with AF responds: *“Yes especially in the beginning, we were thinking a lot about what could happen because you have seen all these movies with cardiac arrest and until you have tried to go to the hospital a couple of times, you are sure that you can die of this.”*

This emerging theme made it apparent that patients and family members had fears and worries about AF that affected their beliefs about the future living with AF.

## 4. Discussion

The current work indicates that not only does AF affect patients, but also the worries and emotions caused by AF may burden close family members. This was expressed in various ways by the family members, who did not always openly share with each other their concerns about AF. Learning to live with AF created a need for the families to understand how the illness manifested itself in daily life, as they would often have to adapt to a new daily rhythm. Families viewed their lives after one of them had been diagnosed with AF as a life filled with feelings of uncertainty about the future, arising from concerns about managing the illness when and if it became worse.

Our findings are consistent with those of other studies, which also found that patients and family members have different ways of coping with AF and that their relationships are being affected by the uncertainty that can be experienced while living with AF [12, 21]. The challenges forced families to develop new strategies. However, for these strategies to be effective, patients and family members must share their emotions and worries [35]. Risom et al. also found that patients with AF could be reluctant to share AF-related psychological distress with family members but that they would often confide in the nurse in a rehabilitation program [35]. Similarly, our study showed that some patients and family members kept their concerns to themselves out of fear of being a burden to each other. Some patients even preferred to visit the doctor alone, which occasionally left family members lacking important knowledge about how to care for their patient. Our findings highlight that it is crucial for healthcare providers to recognise the emotional distress that may be experienced by both patients and their family members. Being confronted with cardiac disease can be stressful, can result in overprotection and negative feelings, and can put pressure on family relationships due to a communication deficit [8]. Furthermore, the theme of *emotional difference* was mirrored in a study that found high levels of anxiety in spouses, which were associated with low perceived health and reduced vitality in their partner and vice versa [9]. Patients and family members affect each other. Therefore, it is important to identify families who need extra support by focusing on the dyadic dynamics influenced by AF. Focusing on the positive resources in the family and the opportunities to make changes in everyday life while living with AF could be beneficial to the health of the patient with AF and the family member.

The theme *changes in family life* revealed how patients and family members were confronted with multiple consequences of AF in their daily lives, both as a family and as individuals. Changes in social activity with friends and family members, changes in mood, and a need for changes to their routine and lifestyle challenged patients and family members to reevaluate the context of their daily lives with AF [36]. Our findings are echoed in studies of patients who have diabetes, stroke, chronic obstructive pulmonary disease, myocardial ischemia, and heart failure and needed to adjust to life with a chronic illness [8, 37, 38]. The REACH-HF trial tested an intervention targeting heart failure patients and their family members [39]. The intervention was a comprehensive, evidence-informed, patient-centred, theory-based self-care support program. Compared with usual care, quantitative caregiver outcomes, like anxiety, did not differ significantly. However, the qualitative data revealed that family members who received the intervention were more likely to make positive changes in how they supported the HF patient, and they also experienced increased confidence in the caregiver role [39]. Involving family members supports the premise that future AF interventions should focus not only on the patient but also on the family members who are required to manage the daily challenges and changes in their routine and lifestyle. Therefore, future AF interventions need to focus on how patients and family members express their need to increase their confidence, knowledge, and capabilities relating to life with AF [40]. Patients in the present study described how the changes caused by AF affected their family structure and roles. Changes in family roles were also found in another study showing that a cardiac event may change family roles as a consequence of cardiac disease [8].

The theme captured in *uncertainty about the future* represents several aspects of patients' and family members' beliefs about how AF may potentially influence their lives. This ranged from a minor to a considerable feeling of uncertainty about the future. Thoughts about how AF could interfere with beliefs about the future were apparent, especially when the patient had just been diagnosed with AF and needed to learn how the manifestations of the illness developed. The theme was similar in families in which a member had a chronic illness as the family needed to familiarise itself with the situation; this is an ongoing process in which the family members cocreate a new context of living with the illness [38, 40]. One study found that being invited to tell their story in encounters with healthcare professionals arranged as family nursing interventions had a positive effect on the wellbeing of patients and family members [37]. Family nursing uses reflexive and therapeutic communication; if conducted in the right way, it can support and empower both patients and family members to be aware of their beliefs, resources, and strategies to cope with AF [36, 40]. Beliefs may be described as the lenses through which we see the world, and they are essential in understanding how families respond to and manage situations that arise from their experience with illness [36]. It is important that nurses understand the patients' and family members' beliefs about an emerging illness, like AF, to enhance wellbeing and healing. Family System Nursing (FSN) recognises that an individual's illness influences the health and wellbeing of their family members and focuses on the interaction, reciprocity, and circularity of beliefs between the patient and their family members [23, 24]. Therefore, giving patients and family members a chance to narrate their illness beliefs and thoughts serves as a way of unburdening oneself, making sense of suffering, and finding hope while living with a chronic illness [15, 16]. Thus, family members are indispensable in supporting self-management and understanding the disease [41]. To systematically involve the patient with AF and their family members would require a shift from patient-centred care to family-centred care through education and supervision of healthcare professionals [42]. A clinical trial using family-focused nursing care for patients with heart failure showed positive results regarding self-efficacy, symptom management, and social activities [43]. Having families narrating their AF stories may, therefore, also contribute to supporting coping with feelings of insecurity and have a positive impact on managing their future with AF. Communication tools to structure conversations with patients and family members and facilitate change may potentially have an effect on the health of patients and family members [16]. However, it requires competence and communication skills, and it requires attention to the family relationships and interventions directed towards the aspects in focus. Furthermore, supporting both the patient with AF and his/her close family member requires that both are willing to communicate and confront their concerns [37]. Our findings indicated that families were missing support interventions targeting both the family members and the patient in order for them to manage everyday life. Family interventions that examine when patients with AF and their family members may benefit from family nursing are warranted.

## 5. Methodological Considerations and Limitations

One of the limitations of this study was that half of the interviewed families consisted of patients and their spouses. Therefore, the study may have focused more on the perspective of living as a couple than on other family constellations. We ended up including patients who were able to attend with a close family member who was a partner. The results might have been different if more patients had participated with friends and family members with whom they were not sharing their everyday lives. On the other hand, this study shows that, in most cases, the patient chose their partner. Furthermore, interviewing only two family members together limits the finding to the experiences of these two family members. However, many of the challenges faced by couples appear to be similar to those of other family members, who may not be quite as close to the patient's everyday life. Another aspect is the number of patients who did not have a family member or refused to participate in the study, which could raise the question whether how these patients experience their everyday life with AF. We chose to focus on the family narrative, studying the family as a unit. Even so, we could also have focused more on analysing the interactions between the interviewed family members, which might have added a more dynamic perspective on how the family members influenced each other and coped with their life situations. Furthermore, we did not focus on coping processes in the family, which could have been a relevant aspect.

The dyadic interviews required ethical considerations to ensure that they were performed in a safe setting when the patient and family member shared their experiences [44]. Therefore, the interviewer had to navigate through the interview, being aware of the patients' and the family members' needs and dedicating them equal attention. The dyadic interview could have negatively influenced the findings if the facilitation of the interviews failed to obtain the patient and family member's experiences. However, we cannot know if some patients or family members moderated their answers even though they responded very willingly to the interview questions. Anyhow, interviews were conducted in the hospital and in the families' own homes, and the variety in contexts appeared to be a strength because we were able to unveil similarities between families regardless of the setting in which the interview was conducted.

Rigour was established by carefully following the method suggested by Giorgi [27] and by following the concepts of trustworthiness in qualitative research: credibility, transferability, dependability, and confirmability. To ensure dependability, quotations were used for transparency in showing the participants' voices [27]. Confirmability was obtained by authors discussing the interpretation of the phenomena of interest several times in an iterative process to strengthen the study's credibility [33]. Throughout the entire process of analysis, all authors aimed to remain open-minded about the phenomena of the lived experiences; they attempted to describe the data as they emerged rather than allowing personal preconceptions or theoretical concepts to navigate or explain the concepts [45]. Transferability was enhanced by revealing the interviewers' preunderstanding and by preparing a transparent description of the different significant elements and the process of study. The findings in this study may also be relevant and important in other contexts, for example, families experiencing acute illness or receiving palliative care.

## 6. Conclusion

The aim of this study was to use the perspectives of the patients and the family members to explore how families live with AF. Three major themes emerged: *emotional differences*, *changes in family life*, and *uncertainty about the future.* These themes captured the essence of how patients and family members experienced their lives living with AF. The study found that patients and their family members saw their life with AF as one fraught with many concerns that caused different emotions that were not always shared. Families did not always share emotions, either to protect each other or out of a lack of knowledge about AF. Furthermore, AF caused changes in how the family planned social life, work, and household activities. Patients and family members talked about their feelings of uncertainty about the future and living with the arrhythmia. In some family members' minds, AF was associated with thoughts about a heart attack or acute stroke. When patients are confronted with AF, the patient and family members should be given the opportunity to talk about issues from a family perspective. In addition to treatment, discussions should focus on concerns about AF, including the risk of severe palpitations and anxiety about the occurrence of a stroke or cardiac arrest. Knowledge about the lived experiences of how families cope with an arrhythmia, like AF, should lead to a shift in clinical practice regarding how healthcare professionals facilitate knowledge and instructions for living with AF. Future research is necessary to explore how nurses and other clinicians may engage in and communicate with patients living with AF and their families.

## Figures and Tables

**Table 1:** Characteristics of patients and family members.

	*N*	Mean (years) (range)
Patients	12	
Female	7	
Male	5	
Age		66 (49–86)
Family members	11	
Spouse	6	
Male friend	2	
Sister	1	
Female friend	1	
Daughter	1	
Without a family member	1	
Age		64 (49–79)
Years since being diagnosed with *∗*AF		3.2 (1.5 months to 22 years)

*∗*Atrial fibrillation (AF).

**Table 2:** Analysis process from derived meaning units to final themes.

Meaning units	Subthemes	Themes
Talking about atrial fibrillation (AF)	Family members have different worries in their everyday life with AF	Emotional differences
We are still two people		
Finding causes		
Why our family		
The first feeling		
Do not be a burden on the family		
Do not be a burden to the hospital		
Using the network	To see life in a new way and find meaning	
Encourage each other		
Changed mood		
Show or hide emotions		

AF intervenes in family activities	Everyday life activities change	Change in family life
Care and protection of children living at home		
Maintain normal daily life		
Changed social relationships	Living with AF affects social life	
Talk about prioritising		
Different limits on how much you can handle		
Get support and motivation for new habits	AF changes the need for knowledge	
Changed lifestyle		
Family knowledge of AF		
Information from AF patients to children Knowledge sharing		

Family thoughts on AF	Fear of how AF will progress influences everyday life	Uncertainty about the future
Getting the right support living with AF		
Support and involvement of family members		
Relatives on alert		
Rituals and strategies		
Fear and myths	AF brings fear of death into everyday life	
Thoughts about the future		
Master the unpredictable		
Thoughts on death		

## Data Availability

All relevant data are within the manuscript.

## Supplementary Materials

Supplementary Materials COREQ (COnsolidated criteria for REporting Qualitative research) checklist.
